# An association of Orf virus infection among sheep and goats with herd health programme in Terengganu state, eastern region of the peninsular Malaysia

**DOI:** 10.1186/s12917-019-1999-1

**Published:** 2019-07-18

**Authors:** Jamilu Abubakar Bala, Krishnan Nair Balakrishnan, Ashwaq Ahmed Abdullah, Lawan Adamu, Muhammad Syaafii bin Noorzahari, Lau Kah May, Hassana Kyari Mangga, Mohd Termizi Ghazali, Ramlan Bin Mohamed, Abd Wahid Haron, Mustapha Mohamed Noordin, Mohd Azmi Mohd Lila

**Affiliations:** 1 Virology Unit, Department of Pathology and Microbiology, Faculty of Veterinary Medicine, Universiti Putra Malaysia, 43400 Serdang, Selangor Malaysia; 2 Microbiology Unit, Department of Medical Laboratory Science, Faculty of Allied Health Sciences, Bayero University Kano, P.M.B. 3011, Kano, Nigeria; 3 Institute of Bioscience, Universiti Putra Malaysia, 43400 Serdang, Selangor Malaysia; 4 Department of Microbiology, Faculty of Applied Science, Taiz University, Taiz, Yemen; 5 Department of Veterinary Clinical Studies, Faculty of Veterinary Medicine, Universiti Putra Malaysia, 43400 Serdang, Selangor Malaysia; 6 Department of Microbiology, Faculty of Science, University of Maiduguri, P.M.B 1069, Maiduguri, Borno Nigeria; 7 Jabatan Perkhidmatan Veterinar Negeri Terengganu Peti Surat 203, 20720 Kuala Terengganu, Malaysia; 8 Institut Penyelidikan Haiwan, (IPH), Veterinary Research Institute, Ipoh, 59, Jalan Sultan Azlan Shah, 31400 Ipoh, Perak Malaysia

**Keywords:** Orf virus, Prevalence, Antibody, Risk factors, Herd-health programme

## Abstract

**Background:**

Orf virus causes a scabby skin lesions which decreases productivity in small ruminants. The unknown status of this disease in the eastern region of Peninsular Malaysia warrants a study to determine sero-prevalence of orf with regards to farmers’ compliance level towards the Herd Health Program (HHP) programme.

**Results:**

Out of 504 animals, 115 were positive for Orf-virus antibodies. An overall prevalence rate of 22.8% indicated a high prevalence of orf disease in this region. It was observed that 25.1% (92/367) of goats were positive and 16.8% (23/137) of sheep sero-converted for Orf virus antibody. Several factors were measured for their possible association with prevalence of Orf virus infection. The prevalence was higher in LY farm, JC breed, kid and female animals, and in the presence of disease lesion. Chi-square analysis showed a significant association of three risk factors which are species, age and sex of the animals (*P* < 0.05). Notwithstanding, all other variables showed no significant difference (*P* > 0.05). Farms surveyed usually practised intensive management system, keeping animals in the shade at all time, due to limited availability of suitable land as a free-range grazing area. An interview with small holder farmers revealed a lack of awareness of the main goals of herd health programme. An overall compliance level of 42.7% was observed for all HHP parameters. Among the 14 main components of HHP modules, animal identification had recorded highest compliance level (84.62%) while milking management recorded the least compliance (− 82.69%). That explained why there was a high sporadic prevalence of Orf infection in this region.

**Conclusion:**

Good herd health supervision is a rehearsal target to prevent an outbreak and the spread of diseases thus reduces economic losses among farmers. Therefore, a good herd health programme should be in place, in order to prevent and control disease transmission as well as to improve herd immunity.

**Electronic supplementary material:**

The online version of this article (10.1186/s12917-019-1999-1) contains supplementary material, which is available to authorized users.

## Background

Small ruminants have important contributions to human kind and sustainability in terms of their meat, milk, and other ornamental products including biological products for studies of diabetes and insulin production [1–4]. However, several diseases such as contagious ecthyma and pneumonic mannheimiasis present a serious challenge and also affects the productive capacities and benefits of these animal species [5]. Contagious ecthyma is a skin disease caused by Orf virus which is otherwise called sore mouth or infectious labial dermatitis. The virus belongs to the family Poxviridae and genus parapoxvirus which is made up of linear double stranded DNA particles [6]. Several species of animals are susceptible to the infection including sheep, goats, dogs, cattle, camels, some wild animal species and human [7, 8]. Nonetheless, contagious ecthyma is considered primarily a disease affecting goat and sheep population worldwide [9, 10]. The disease is also a very important zoonotic viral infection associated with painful non systemic proliferative lesions [11, 12]. Rarely, long standing infections can become complicated by secondary bacterial infection that may extend to internal organs [13]. Transmission occurs by direct contact with an infected animal and/or with contaminated fomites that contain orf virus. Traditionally, the virus will enter the skin through cuts or abrasions and establish the infection. The skin lesions develop and progress in multiple stages ranging from skin reddening (erythema), macule, papule, vesicle, pustule, scab and scar [14, 15]. Contagious ecthyma usually resolves spontaneously, however in severe cases due to secondary infections or delayed nursing intervention, the economic impact is significant due to deaths and wasting. Similarly, majority of human infections are localized and heal spontaneously without much complications; however, immunocompromised patients can develop large, poorly healed lesions.

The best method for diagnosis of Orf virus infection is culture of the virus on susceptible cell lines [16–18] but this is mostly characterized as laborious and time-consuming [17]. Moreso, molecular detection using specific Orf virus has been developed, but such assays are unlikely to be useful for herd screening [19]. Enzyme Linked-Immunorsorbent Assat (ELISA) method can be sensitive and inexpensive for field application in the detection of Orf viruses from large number of animal population.

At present some commercial vaccines can control this disease however, anecdotal epizootics of a persistent generalized form of the infection has been reported among goats vaccinated with the first generation orf vaccine prepared for sheep [20]. Similarly, outbreaks of more virulent contagious ecthyma has also been reported among goats vaccinated with the goat-derived contagious ecthyma vaccine [21]. Furthermore, these vaccines have been reported to be associated drawbacks including the inability to produce effective and desirable protection of the vaccinated animals, as well as persistent virus shedding into the environment which poses an increased risk to other susceptible animals [22]. Therefore, vaccination against Orf virus is only recommended in endemic areas [15]. The urgent need to develop an effective vaccine against contagious ecthyma is borne out of its economic importance to especially rural farmers as well as its zoonotic potentials particularly among animal handlers.

Many outbreaks of contagious ecthyma disease in different part of the world including but not limited to Africa, Middle East, Europe, North America and most of the Asean countries including Malaysia [7, 22, 23] thus it has have become a major concern due to the huge economic losses. According to Onyango et al. [24] the estimated national costs of orf disease in the British sheep industry based could reach up to a staggering ₤10 million. Moreover, livestock rearing is ranked the highest practice among other agricultural sub-sectors worldwide particularly in Asian regions including Malaysia. A report by the Malaysian Agricultural analysis and Development Institute (MARDI) revealed that small ruminants are among the most popular livestock farming practiced in Malaysia and it is estimated to be 280 times more than the poultry industry. However, this promising industry is being challenged by the menace of infectious diseases including contagious ecthyma which has resulted in a huge financial loss in the country [25]. A recent works conducted by Sadiq et al., [9], Jesse et al., [26] and Bala et al., [27] have elucidated on the prevalence of Orf in the state of Selangor, Malaysia which poses an alarming prevalence high prevalence. However, adequate information on Orf outbreak is lacking on eastern region of Peninsular Malaysia thus, the associated risk factors including the impact on sheep and goat husbandry practices is still obscure. The present study was conducted to determine the seroprevalence of Orf virus in Terengganu state. In our knowledge, this has given the idea that serves as the first documented report on the prevalence of contagious ecthyma in this region. Moreover, this study have further elucidated on the farmers’ level of compliance with established herd health programme. A well thoughtful of the current epidemiological situation of Contagious ecthyma infection in the state of Terengganu and Herd health management would allow the establishment of improved disease control program that would benefit small holder farmers.

## Results

All the 13 farms surveyed have consented and responded to the questionnaire. A total of 504 blood samples were collected from sheep and goats.

### Serological assay

Out of the 504 animals sampled, one hundred and fifteen (115) were positive for orf virus antibody based on the ELISA assay. This indicated an overall score of 22.8% among the animals sampled across the 13 farms selected. Goat population had the highest percentage (25.1%) of sero-conversion compared to sheep with 16.8% positive results. The finding is statistically significant with *P*-value of less than 0.05 (Table 1).

**Table 1:** Overall result of ELISA according to animal species

Species	Total sample (n)	Negative	Positive	Sero-conversion rate (%)	95% Confidence interval		*P*-value
					Lower	Upper	
Goat	367	275	92	25.1	0.2091	0.2975	0.0437
Sheep	137	114	23	16.8	0.1146	0.2393	
Total	504	389	115	22.8	0.1937	0.2679	

### Cross-reactivity, sensitivity and specificity of ELISA assay

There is no cross-reactivity observed upon testing with other related viruses including bluetongue virus (BTV) and Schemallenburg virus (SBV) positive sera samples obtained from sero-converted infected animals. Both BTV and SBV postive control sera produced an optical density (O.D) value below the cut-off reading. Noteworthy, based on the sensitivity (Se) and specificity (Sp) obtained from ROC-curve analaysis determined using MedCalc sortware, the ELISA presented an Se and Sp of approximately 95.2 and 97.8% respectively showed by the area under the curve (AUC).

### Rate of sero-conversion according to sample farms

Table 2 depicted level of antibody titre for orf virus in relation to sampling location. The highest sero-conversion rate was observed in farm LY (60%), followed by farm LZ (41.7%) and the least was evident in farm LM (12%). The association among farms and prevalence rate of orf virus infection is not significant Chi square ((X^2^) = 17.889; *P* = 0.1191).

**Table 2:** Sero-converted animals according to sample farms

Farm	Total sample (n)	Negative	Positive	Sero-conversion rate (%)	95% Confidence interval	
					Lower	Upper
AB	49	39	10	20.4	0.1148	0.3364
BF	35	28	7	20.0	0.1004	0.3589
LB	11	9	2	18.2	0.0514	0.477
LH	19	14	5	26.32	0.1181	0.488
LI	105	85	20	19.1	0.1268	0.276
LM	25	22	3	12.0	0.0417	0.2996
LN	22	14	8	36.4	0.1973	0.5704
LS	17	12	5	29.4	0.1328	0.5313
LW	33	25	8	24.2	0.1283	0.4102
LY	5	2	3	60.0	0.2307	0.8824
LZ	12	7	5	41.7	0.1933	0.6805
MF	83	57	26	31.3	0.2237	0.4195
PT	88	75	13	14.8	0.0884	0.2365
Total	504	389	115	22.8	0.1937	0.2668

### Rate of sero-conversion according to breeds of animals

Table 3 depicted rate of sero-conversion for orf virus in relation to breeds of animals. The highest rate was recorded among Jamnapari cross (JC) breed (50%), followed by Saanen breed (27.6%) and the least was found among Boer (BO) and Toggenburg (TO) both had 0.0%. The association amongst the farms and prevalence rate of orf virus infection is not significant (X^2^ = 17.093; *P* = 0.0723).

**Table 3:** Sero-converted animals according to breeds

Breed	Total sample (n)	Negative	Positive	Sero-conversion rate (%)	95% Confidence interval	
					Lower	Upper
BB	88	75	13	14.8	0.0884	0.2365
BC	46	37	9	19.6	0.1065	0.3318
BO	4	4	0	0	0.0000	0.4899
DO	49	39	10	20.4	0.1148	0.3364
JC	2	1	1	50.0	0.0945	0.9055
JP	98	79	19	19.39	0.1278	0.2831
KC	3	3	0	0	0.0000	0.5615
KJ	56	35	21	37.5	0.2601	0.5059
SA	105	76	29	27.6	0.1997	0.3685
SC	52	39	13	25	0.1523	0.3821
TO	1	1	0	0	0.0000	0.7935
Total	504	389	115	22.8	0.1937	0.2668

*BB* Barbados Blackbelly, *BC* Boer Cross, *BO* Boer, *DO* Doper, *JC* Jamnapari Cross, *JP* Jamnapari, *KC* Katjang Cross, *KJ* Kajang, *SA* Saanen, *SC* Saanen Cross, *TO* Toggenburg

### Rate of sero-conversion based on risk factors

The highest prevalence of orf disease was found among kids of less than 3-months old. All of them were sero-converted. This was followed by 29.7% in animals of older than 4 years. Interestingly, animals aged 4–9 months have the lowest positive rate (20%). The association amongst the various age groups and rate of sero-coversion for orf virus infection is significant (X^2^ = 8.163; *P* = 0.0428). The association amongst the various age groups and rate of sero-conversion antibody for orf virus infection is significant (X^2^ = 8.163; *P* = 0.0428).

From a total of 174 male sheep and goats that were examined, 31 (17.8%) were sero-converted for Orf virus. However, out of a total of 330 female sheep and goats examined, 84 (25.5%) were sero-converted for the virus (Table 4). The association amongst the gender of animals and the rate of sero-conversion for orf virus is significant (X^2^ = 3.886; *P* = 0.0487).

**Table 4:** Sero-converted animals according to other risk factors

Variable	Total sample (n)	Negative	Positive	Sero-conversion rate (%)	95% Confidence interval	
					Upper	Lower
Age Months						
Kid (0–3)	2	0	2	100	0.3424	1.0000
Young (4–9)	105	84	21	20	0.1347	0.2865
Adult (10–36)	333	260	73	21.9	0.1781	0.2667
Old (37–72)	64	45	19	29.7	0.1991	0.4177
Gender						
Female	330	246	84	25.5	0.2105	0.3041
Male	174	143	31	17.8	0.1285	0.2418
Orf Lesion						
Absent	501	388	113	22.6	0.1911	0.2641
Present	3	1	2	66.7	0.2077	0.9385
History of orf						
No	504	389	115	22.82	0.1937	0.2668
Yes	0	0	0	0	0.0000	0.0000
Vaccine against orf						
No	504	389	115	22.82	0.1937	0.2668
Yes	0	0	0	0	0.0000	0.0000

The rate of sero-conversion among animals with clinical orf disease (66.7%) was higher than the animal’s who are devoid of obvious clinical manifestations of orf (22.6%). The association between the occurrence of clinical orf disease and sero-conversion is not significant (X^2^ = 2.629; *P* = 0.1049).

Among 504 sheep and goats sampled, none of the farms were observed to practice Orf vaccination to their animals. Therefore, no association exists between the vaccination history and the sero-conversion rate of Orf disease amongst all the farms as the potential risk factor.

### Result for HHP farmer’s compliance level

**Fig. 1:**
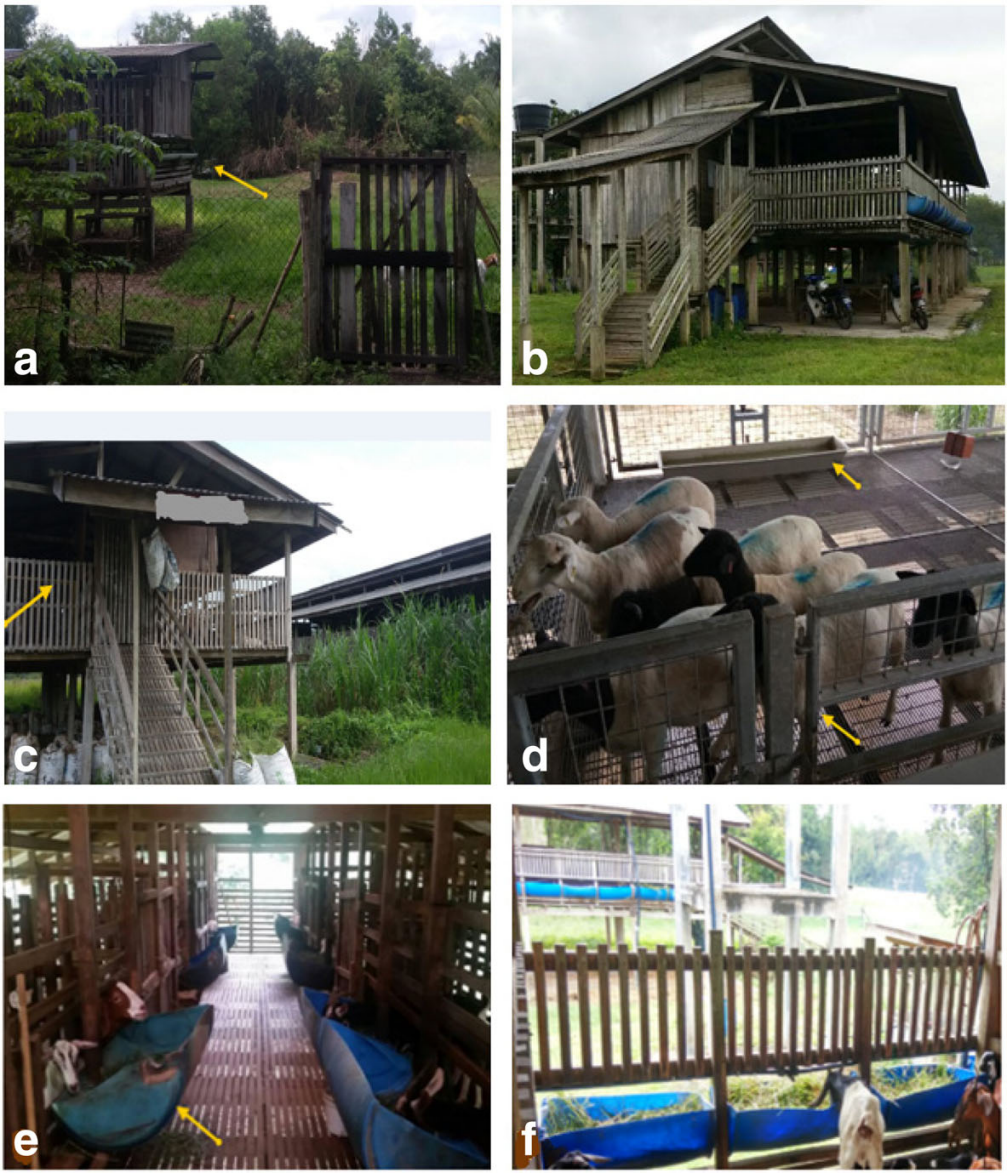
Housing condition, roofing, flooring, ventilation, sanitation, Feeds and feeding management. The flooring type is wooden and elevated (**a**, **b**, **c**). Some may use metal fence with wooden frame and wooden flooring (**d**), Feeding utensils (**e**); and (**f**) pellets and grasses that for the sheep and goats

All farmers raised either pure breed or crossbred Boer, Boer cross, Katjang, Katjan cross, Jamnapari, Jamnapari cross, Saanen, Saanen cross, Barbados Blackbelly, Doper, and Toggenburg breeds. Majority of the farms raise their sheep and goat primarily for meat, however, rearing for dairy purposes have been considered a convenient alternative. Animals were reared in a simple shed with low-cost housing materials. Animals were fed with cut-grasses and feed pellets under a standard ration. Farmers employed a strong rubber made feeding utensils for feeding. Drinking water were provided ad-libitum. Trees for shading and grasses for animal feed were grown in the pasture areas nearby. Figure 1 shows the type of housing, the type of feed and feeding system generally observed in most farms.

**Fig. 2:**
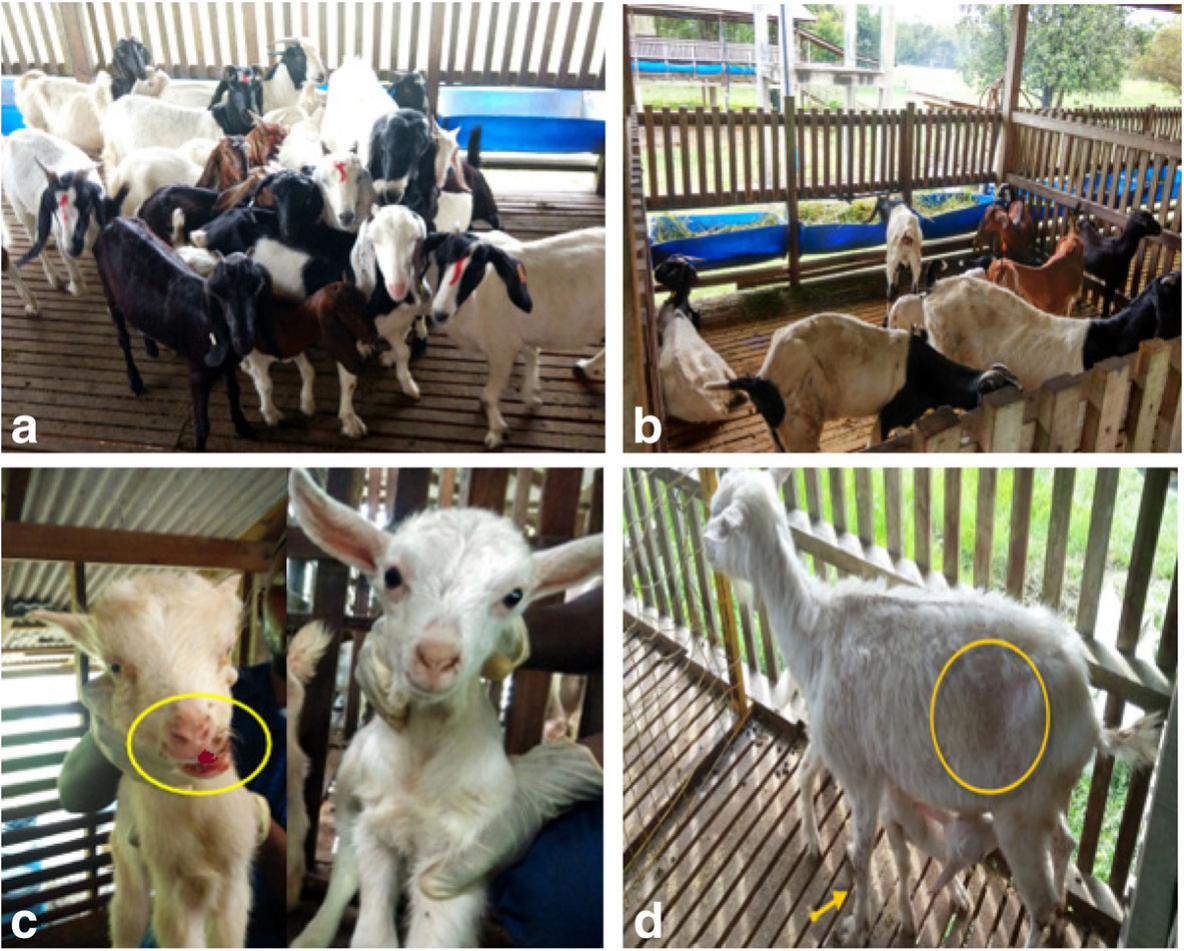
Animal condition inside the pens of the intensively managed farms: healthy animals (**a**, **b**); and sick animals in isolation (**c**, **d**) with clinical disease and orf skin lesions (as indicated in the photography)

Figure 2 depicted the general body condition of some of the animals surveyed. Both sheep goats were continuously kept under this housing confinement with limited access to grazing and pasture areas. The overall compliance level based on the total 93 questions answered by the farmers is 42.7% while an overall non-compliance level observed was 57.7%. Table 5 showed the farms level of compliance and non-compliance to HHP’s modules, Farms AB, PT and LM showed the highest compliance level with 15, 14 and 14% respectively while, farms LY, LS, LZ, and LN recorded the highest non-compliance level of 12, 11, 11, and 11% respectively to HHP modules.

**Table 5:** Respective compliance level of farms’ herd health program

Farms	Compliance (%)	Non- Compliance (%)
LS	3	11
PT	14	3
AB	15	2
LM	14	3
BF	7	9
LI	11	5
MF	8	8
LH	8	8
LZ	3	11
LB	6	9
LW	6	9
LY	3	12
LN	3	11

Table 6 presented the summary of the overall farm compliance level to HHP modules as analyzed by nominal regression analysis. Farms LS, PT, AB, LM, LZ, LY and LN showed strong compliance (*p* < 0.0001) for HHP. However, the effect of HHP modules on farms BF, MF, LH, LB and LW did not record a significant compliance level (*p* > 0.05); indicating decrease compliance level by these farms to parameters of HHP modules. The overall effect likelihood ratio of HHP program on various farms compliance levels is strong and significant (X^2^ = 302.61; *P* < 0.0001).

**Table 6:** Compliance level to herd health program

Farms	Test Estimates	Standard error	Chi square value	*P*-value	95% Confidence Interval	
					Lower	Upper
LS	−0.3433	0.0686	25.02	< 0.0001	−0.4786	−0.2093
PT	1.5754	0.2382	43.73	< 0.0001	1.1248	2.0632
AB	1.9145	0.2619	53.44	< 0.0001	1.4256	2.4588
LM	1.7029	0.2463	47.79	< 0.0001	1.2392	2.2100
BF	−0.2079	0.2096	0.98	0.3212	−0.6266	0.1976
LI	0.7577	0.2066	13.45	0.0002	0.3565	1.1688
MF	0.1056	0.2040	0.27	0.6048	−0.2981	0.5039
LH	0.0619	0.2045	0.09	0.7623	−0.3433	0.4601
LZ	−1.2280	0.2619	21.99	< 0.0001	−1.7722	−0.7390
LB	−0.3987	0.2153	3.43	0.0640	−0.8314	0.0154
LW	−0.3499	0.2137	2.68	0.1015	−0.7787	0.0617
LY	−1.4738	0.2835	27.03	< 0.0001	−2.0704	−0.9501
LN	−1.3054	0.2683	23.68	< 0.0001	−1.8650	−0.8061

**Fig. 3:**
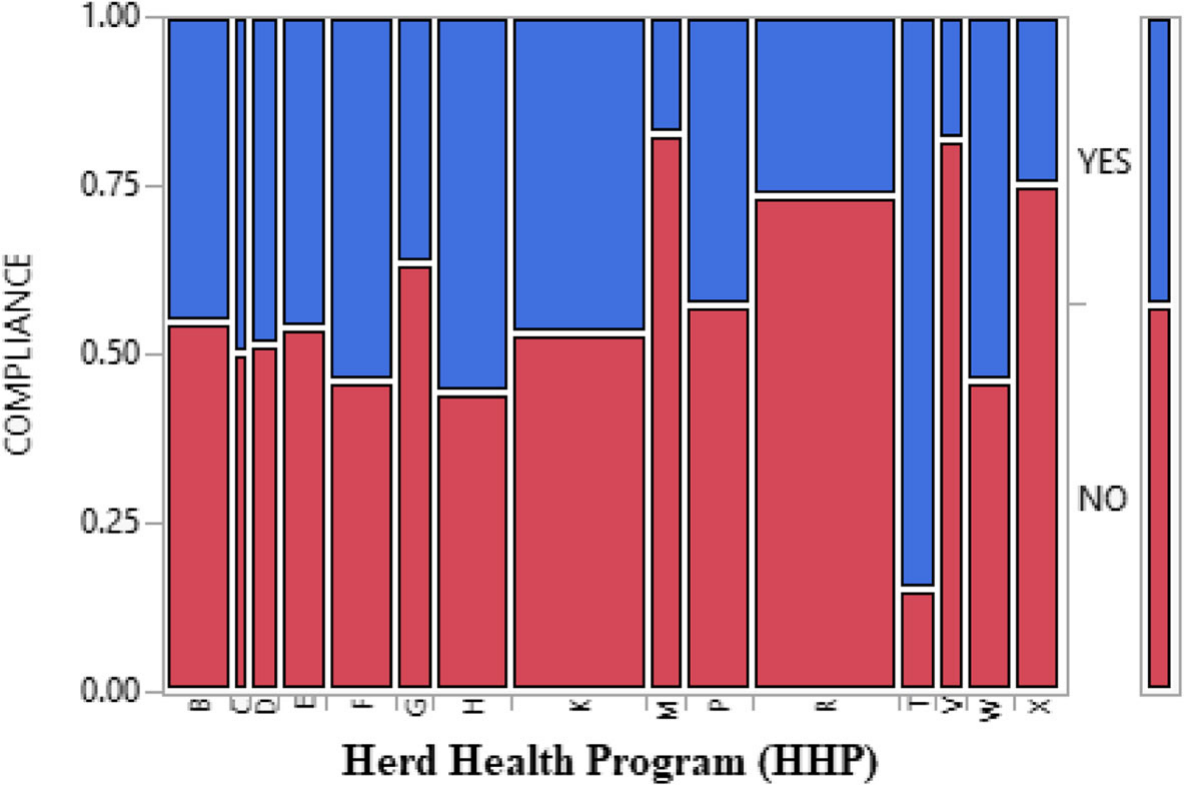
Mosaic plot presentation of the main components of HHP modules and compliance level

Table 7 presents the main components of HHP modules and the levels of compliances and non-compliances. Animal identification (T) recorded highest compliance level of 84.62%, followed by housing condition (eg: roof, flooring, ventilation, sanitation) (H) with value of 55.77% compliance level; moreover, milking management (M) recorded the highest level of non-compliance of 82.69%. The association amongst the main components of HHP modules and the level of compliances and non-compliance is strong and significant (X^2^ = 114.77; P < 0.0001). Mosaic plot presentation of the main Components of HHP Modules and compliance level is shown in Fig. 3.

**Table 7:** Main components of HHP modules and the level of farmer’s compliance and non- compliance

HHP module	Total Number of Answers	Level of Compliance	Level of Non-Compliance	Chi-Square and P-Value
H	104	58 (55.77%)	46 (44.23%)	**X**^**2**^ **= 114.77** ***P*** **< 0.0001**
F	91	49 (53.85%)	42 (46.15%)	
P	91	39 (42.86%)	52 (57.14%)	
D	39	19 (48.72%)	20 (51.28%)	
V	39	7 (17.95%)	32 (82.05%)	
B	91	41 (45.05%)	50 (54.95%)	
W	65	35 (53.85%)	30 (46.15%)	
C	26	13 (50%)	13 (50%)	
M	52	9 (17.31%)	43 (82.69%)	
R	195	52 (26.67%)	143 (73.33%)	
K	182	85 (46.70%)	97 (53.30%)	
E	65	30 (46.15%)	35 (53.85%)	
T	52	44 (84.62%)	8 (15.38%)	
G	52	19 (36.54%)	33 (63.46%)	
X	65	16 (24.62%)	49 (75.38%)	
Total	**1209**	**516 (42.68)**	**693 (57.35)**	

All the abbreviation were defined in Table 8 above

**Fig. 4:**
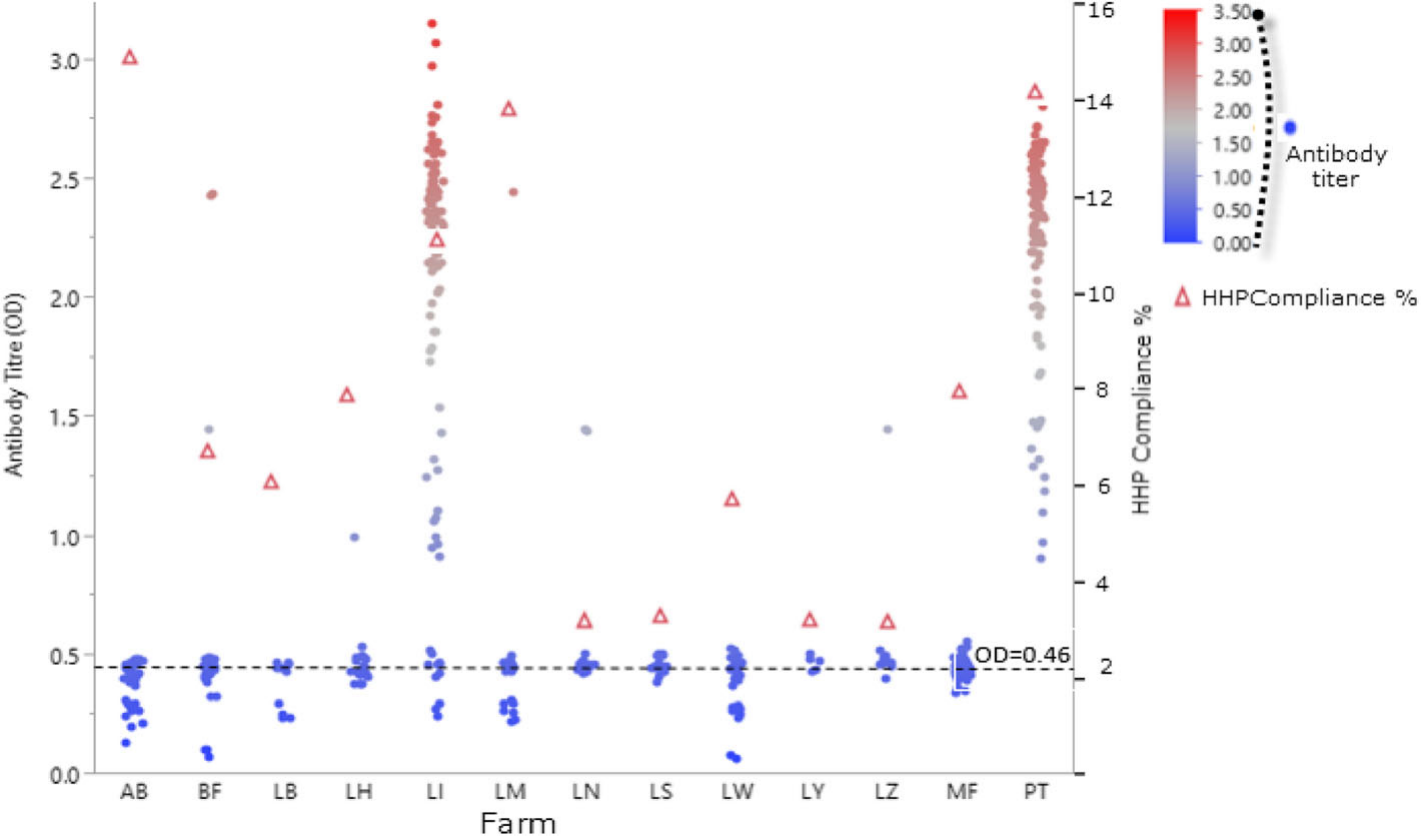
Unequal distribution of antibody titres (based on OD reading) against orf virus among animals in the respective farms. Each data point in the graph represents antibody titre of individual animal. The horizontal line (−--) represents cut-off point of OD for negative sera (negative results). HHP compliance level (in %) for the respective farms

Figure 4 depicts the distribution of mean antibody titre among all the farms sampled. Farms LI and PT have the highest antibody titres, whereas farm LW have the lowest antibody titres. Majority of the antibody titres were below OD reading of < 0.5.

## Discussion

This investigation reported the current status of orf virus disease in the Terengganu state, eastern coast region of Peninsular Malaysia. The study revealed evidence of the presence of orf virus infection among the sheep and goats’ population as confirmed by ELISA assay which amounted to a prevalence of 16.8% in sheep and 25.1% in goats. Replication of Orf virus occurs in the epithelial cell surfaces of the skin layers and at the mucosa of the mouth, oesophagus as well as hairless parts of the body serving as primary sites of the lesions. Accidental abrasions of the skin due to hard stubble, thistles or any analogous plant promote access of the virus and initiates the replication cycle [28–31]. Upon successful entry and attaining the incubation period of approximately 10 days the disease is disseminated to the other tissue and host’s response is mounted and therefore this will lead to the production of antibodies that could be detected in body fluids such as blood, saliva and mucosal secretions [22, 32]. Several studies have stated that clinical orf virus symptoms are first seen in the fourth and fifth days of exposure, whereas the levels of anti-orf virus specific antibodies are normally detectable between eighth and tenth days of exposures to the virus.

ELISA test employed in this study revealed an overall seroprevalence rate of 22.8% based on antibody titre among the sampled population. This indicates a considerably high sero-conversion rate of the disease compared with a similar study in the Selangor, Malaysia with a prevalence rate of 14.4% in goats and 12.2% in sheep [27]. However another report by Jesse et al. [26] which was high prevalence rate (36.7%) among goat population, based on IgM detection observed and thus indicates an active infection in that State. Generally, it is suggested that orf disease is a serious issue in Malaysia which is recurring frequently at an alarming rate in different parts of the country [26]. Similarly, consistent with our findings, the prevalence of orf in other parts of the world is high [22]. Orf infection reported was 19.51% among lamb in England [24], 34.89% in China [11], staggering 98% Nilgiri Hills in Tamil Nadu, India [33] and 54% in Saudi Arabia [34]. Gökce et al. [28] had also reported a high sero-conversion rate of 52.8% among lambs within some selected districts in Turkey. The high morbidity of this disease underscores the infectious nature of this virus and its economic impact on the goat industry [9, 22, 35]. It has been speculated that contagious ecthyma disease of sheep does not confer long term protection thus, seasonal outbreaks among herds are common [36, 37]. Shed viruses from animals remains viable for decades as such they served as source for the sporadic spread of the virus in the same herd. Thus resulting in the ultimate transmission of the virus to neighbouring herds via transporting of infected animals.

Various putative risk factors for the prevalence rate were examined to ascertain the possible risk factors. Identification of relevant risk factors is crucial for the proper disease management and outbreak containments. We identified species, age and sex of the animal to be the most significant risk factors. Goat species recorded highest prevalence of 25.1% compared to 16.8% that of ovine like the report by Jesse et al. [26]. Naturally, goats are naturally more aggressive than ovine, hence they tend to cause injury among themselves leading to higher susceptibility to orf virus transmitted via direct contact. Additionally, most small holders do not practice dehorning for their animals, as such this may subject the animals to injuries due to fighting. Animals could easily get wounded, cuts and abrasions as a predisposal factor for virus penetration via a wounded skin [38–41].

Similarly, female gender had the highest prevalence and this was recorded as a significant risk factor in this study. A similar observation was reported by Orgeur et al. [38]. More aggressive behaviour of males expected to contribute higher number of cases. However, unequal sample size by which majority of the subjects studied were female may contribute to our unparallelled observation. Meanwhile, previous observations showed that orf infection have not discriminatory tendencies between gender [22, 42].

Among the 13 farms, farm LY showed the highest historical orf virus infection. This is strongly associated with the fact that farm LY recorded the highest non-compliance levelof HHP. A strict adherence to HHP modules shall reduce the general risk of exposure to contagious ecthyma disease in animal population [43, 44]. Animals are susceptible to orf virus infection regardless of their age. There is a moderate morbidity rate of approximately 50% in the affected farms observed in this study, however, the mortality rate of approximately 1% was identied in the affected farms. Interestingly, higher seroprevalence (100%) was observed among kids younger than 3 months of age in comparison to animals older than 4 months, however, this high seroprevalence among the kids has not been associated with high mortality, therefore a more detail confirmatory test such as virus culture and isolation would further distinguishes active infection that neccesiates for vaccination. Furthermore, we do not observed a very a high significant difference between the flocks show clinical disease and the high seroconversion. A similar phenomenon was observed by Bora et al. [36] who studied the prevalence of contagious ecthyma among goats in Assam, India. This observation is attributed to the fact that older animals developed better protective immunity against recurrence orf infection.

Other risk factors examined such as the presence lesion and abrasion, history of clinical orf infection in farms and vaccination practice did not appear to be a significant determinant of orf disease prevalence. The presence of skin lesions usually indicates current infections which are best diagnosed by standard virus isolation and identification [7, 45]. Antibodies produced by animal hosts as a part of either primary or secondary immune response and detected during the latter part of an infection. It may take 1 to 4 weeks following an infection before antibodies can be detected and assayed in ELISA test. Animals with previous exposure to orf virus may carry the virus in their hide or dried scabs and shed the virus into the surrounding environment. Orf virus had a higher survivability in the environment especially in tropical climate [46]. In an endemic environment, similar to other viral diseases, animals are often re-infected with orf especially when they become immunosuppressed [22, 47]. Additionally, previous orf infection does not provide a long-lasting immunity against orf but instead it provides farmers an experience to deal with subsequent infections. Animals that were reinfected often recovers faster compared to the first exposure and shows less severe lesions [46]. Vaccination had been a main prophylactic measure against Orf. Another issue is immunity induced from vaccination which can only last for about 6 months. Booster doses of vaccine were required especially for farms located in areas endemic with orf [48–51].

The HHP module analysed in this study contains variables that are pertinent to epidemiology of orf disease as previously described elsewhere [24, 52, 53]. Unfortunately, all the farms surveyed did not incorporate orf immunization as a part of their HHP. Vaccination of already infected animals has been found to reduce the course and severity of the disease [54]. Our results also indicated that orf virus infection is widespread in the areas of Terengganu State as such vaccination should have been advocated on a regular basis. Even though, some authors are in the opinion that a vaccine against orf disease should not be attempted in herds that do not have previous history of the disease since only live vaccines are available [55]. However, newer effective potential vaccine tried in some experimental animal revealed promising results, it is therefore advisable to vaccinate animals against orf regardless of previous outbreak in the farms [27, 50].

The general objective of HHP is to enhance the herd efficiency through general farming, nourishment administration, vaccination, ecological management and parasite control. It endeavors to arrange all data appropriate to goat crowd wellbeing into a straightforward, usable, and effectively recalled lists. Therefore, a proper record-keeping must be in place to enable and ensure the success of HHP [43, 44]. Vaccination is important to shorten the duration of disease transmission and to confine infections from being spread from animal to animal. As indicated by Steven and Jeremy [56], vaccination programs are intended to contain future infections in the flock and ought to be implemented together with neighboring farms. In Malaysia, the present vaccination experience is targeted against FMD and pneumonia which was not in practice by all farmers in the survey. Our study had successfully and thoroughly surveyed the existing HHP at the farms. An overall compliance level of 42.7% observed is lower than that previously reported by Abdullah et al. [43] on several farms with a compliance level of 56%. Good HHP supervision is a rehearsal target to prevent the expansion and spread of diseases thus reduces economic losses among farmers [57].

Disease monitoring is concerned with understanding changes in its endemicity and distribution [58]. Proper biosecurity measures such as foot dip, vehicle spray, use of proper boots and attire in the farms were not implemented. Interview with small holder farmers also revealed a lack of awareness of what are the main goals of herd health programme. Farms surveyed usually practised intensive management system due to limited availability of land to rear their goats. This caused the likelihood of animals contracting orf virus infection due to an increase of crowding among animals [27, 32].

Among the 14 main components of HHP modules, milking management is observed to have the highest level of non-compliances (82.69%), whereas, animal identification had recorded highest compliance level of 84.62%. The highest compliance level associated with animal identification indicated an appreciable livestock production is in accordance with the standard recommended by the OIE. Herd-health information is pertinent to livestock producers and to the public as well as animal welfare, fully comprehending of types and sources of animal’s heath that farmers will utilized is very vital [59].

Many studies carried on the relationship between dose antibody responses and virus replication have generated controversies [60–63] The level of humoral immune response against the virus is directly related to the level of virus replication achieved [64, 65]. Titers of antibodies are normally positively correlated with the level of total virus binding antibody titers [66–68]. Therefore, it shall be a significant association amongst total virus production in the host and the humoral responses of antibody titres as directed against important viral disease such influenza [63]. However, in many cases, high antibody titre does not necessarily translate into protection against re-infection and previously exposed animals to orf viruses do not necessarily enjoy protection against re-infection [69]. We noted that despite the high compliance level to HHP, many animals in farms LI and PT had high antibody titres against orf virus. Due to the fact that no vaccination against orf virus was given and no current clinical infection was observed, it is suggested that the cohort group were suspected to have recovered from recent infection following an increase in antibody titres against the virus. The vaccination shall contribute the most significant weightage towards the success of HHP entirely. In addition, watchful understanding of the different types of protective immunity against orf virus is important for the development of a safer vaccines and containment of virus spread.

Control and prevention of Orf disease is important to ensure it does not widely spread in the entire animal population [70–73]. A viable animal health program is a fundamental piece of an effective animal production, herd heath program (HHP) is an essential tool for monitoring disease for prevention and control programs [43, 56]. Legitimate sustaining and rearing won’t bring about most extreme generation if goats are not healthy, there’s a scarcity of knowledge concerning the farmers’ compliance level on correct herd health program practiced by the livestock farmers in Malaysia. This information is very important to increase the productivity of the farms and for future development of temporary herd health programs for small ruminant farms.

## Conclusions

Interaction between virus and body immune system is a battle among the parties (virus and host). In primary orf virus disease, the virus replicates in the epithelial cells for a period before the host can mount an effective immune response. This leads to an appearance of IgG or IgM that were incriminated as a signal of orf infection. The overall prevalence was found to be 22.8% (25.1% in goats and 16.8% in sheep). Significant risk factors identified were specie, age, and sex. A higher sero-conversion rate was seen among kids younger than 3-months-old and female gender showed higher antibody titre. Poor implementation of HHP may also be associated with a higher sero-conversion rate of orf virus infection observed. Therefore, it is important to carry out epidemiological surveys [74, 75] in circumstances where there is a risk of introducing disease into a new herd through replacement of sheep from unknown premises. Based on our findings, it will be recommended that lambs in the region should be regularly vaccinated to reduce the severity of Orf and its consequential financial implications, along with routine vaccination, periodic surveillance could be enacted to determine the both temporal and spatial distribution of Orf viruses.

## Methods

### Informed consent and ethical consideration

All the procedures involving animal subjects were conducted in compliance with the recommendations of the Institutional Animal Care and Use Committee (IACUC) – UPM/IACUC/AUP-U013/2018. Goats and sheep from thirteen (13) farms were selected among the private and government owned farms at 4 districts of the Terengganu state. The sampling farms were selected on the basis for the availability of adequate study animals and diversity in agroecology of the areas. Terengganu State is divided into eight (8) administrative districts called *Daerah* in Malay language. The sampling was strategized to capture 4 out of the 8 eight administrative districts as the representative of this state and a total of 13 farms were selected based on the simple random sampling technique. Consent from all participating farms were obtained through written permission of the owners and witnessed by the Terengganu State division of the Department of Veterinary Service (DVS).

### Questionnaire and data collection

A well-structured questionnaire which contained information on farm management practices, possible risk factors and herd health programme implemented by farm owners were filled via an interview session. The questionnaire was designed to contain three (3) sections, namely; Section A (farm management practice), Section B (farm’s HHP compliance level) and Section C (demography and risk factors for exposure of individual animals). The questionnaire template was added separately in the Additional file 1.

### Farm data collection

Section A of the questionnaire which relates to informations on the sampled farms was administered. The relevant data sought included; details of the operator, category of farmer, man-power, annual production, type of housing and management system, as well as population. Section B on the other hand (farmer’s compliance level to HHP), contains questions relating to the farmer’s awareness, compliance level, and knowledge of each of the 14 modules of herd health programs, based on the Department of Veterinary Service, Malaysia (Table 8). Lastly, section C of the questionnaire contains the information on the demography namely; age, sex, and breed, together with information on the putative risk factors such as cut and abrasion on the animal, presence of orf lesion and history of vaccination against orf or any related viral disease.

**Table 8:** Herd health program modules

S/N	Main component of HHP Module	Notation Acronym	Sub-questions	Total number of Questions
1	Housing condition (eg: roof, flooring, ventilation, sanitation)	H	H1 to H8	8
2	Feed and feeding management (feed storage, amount of feed required per animal)	F	F1 to F7	7
3	Parasite control program			
	a. deworming program	P	P1 to P7	7
	b. deticking	D	D1 to D3	3
4	Vaccination program	V	V1 to V3	3
5	Farm biosecurity	B	B1 to B7	7
6	Waste disposal	W	W1 to W5	5
7	Fly, pest and odour control	C	C1 to C2	2
8	Milking management (mastitis control program)	M	M1 to M4	4
9	Reproductive management	R	R1 to R15	15
10	Kid/lamb management	K	K1 to K14	14
11	Doe/ewe management	E	E1 to E5	5
12	Animal identification	T	T1 to T4	4
13	Medication/Drug management (record system, storage)	G	G1 to G4	4
14	Disease monitoring program	X	X1 to X5	5
Total main HHP modules 14		Total number of questions 93		

### Sampling of farms

This investigation involved thirteen (13) sheep and goat farms located at the four major districts namely; Kuala Terengganu, Kuala Nerus, Marang and Setiu in the Terengganu State, East Malaysia. The respondents were given the questionnaire; a response to each question is a dichotomous outcome as either “YES” or “NO”. Where “YES” denotes the farmers’ compliance to that segment of HHP module, while “NO” is otherwise.

### Individual animal data collection

A Thorough physical examination to identify infected animals based on the clinical signs of erythema, papule, vesicle, or pustule around the lip, gums, mouth and tongue and the general body part was conducted. Relevant demographic data from each animal was also recorded in the data sheet prior to sampling. A total of 504 sheep and goat’s samples were collected using simple random sampling method after calculating the sample size according to the standard formula [76, 77]. The formulae and the sample size calculation were as in shown below. After sample collection all the involved animals were closely monitored regularly to avoid any spread of the disease.

$$ \mathrm{n}=\frac{Z^2 pq}{L^2} $$

where, n = sample size

Z = Standard normal distribution at 95% confidence interval = 1.96

p = Prevalence in similar work

q = 1 – p

L = Allowable error, taken as 5% = 0.05

In this study, *P* = 36.7% (Jesse et al., 2018a)

$$ \mathrm{n}=\frac{Z^2 pq}{L^2}=\frac{(1.96)^2\times 0.367\times \left(1-0.0732\right)}{(0.05)^2}\approx 500\ \mathrm{samples} $$

### Preparation of serum sample

Whole blood samples were collected from all the 504 animals in the sampling farms as. A method of collection via jugular venepuncture using a 21 gauge vacutainer needle was applied and collected into a plain serum collection tubes. The tubes containing the whole blood samples were then stored in a cooler box and transported to the laboratory for analysis. The blood samples were left to clot and centrifuged at 3,000 revolution per minute (rpm) for 5 min to separate the serum from the blood. The serum was then pipetted into a 1.5 ml microcentrifuge tubes and stored at -20 °C until required for assay.

### Preparation of hyperimmune positive and negative sera against the pure Orf virus

Hyperimmune serum (HIS) against the UPM1/14 Orf virus isolate was prepared according to the method described by [34, 36, 78–80]. For this purpose, specific anti-Orf virus antibodies were prepared in two healthy goats of about 1 year old. Blood sample were collected as the negative control prior to the start of the procedure. The purified virus suspension contained in DMEM medium was first heat inactivated by incubation at 56 °C for 30 min. One mL of the pure Orf virus antigen was mixed with Complete Freud’s Adjuvant (CFA) (GIBCO BRL, USA). An emulsion containing equal volume of pure virus and CFA was formed by homogenization until a good mixture was obtained. The emulsified suspension was allowed to settle at 4 °C. One goat was then injected subcutaneously with 0.5 mL of this emulsion while the other goat was kept as control. Two weeks later, the goat was re-injected with the same dose of antigens that was emulsified in Incomplete Freund’s Adjuvant (IFA) (GIBCO BRL, USA). The injections were then repeated weekly for 4 weeks. One week after the last injection, the goat was injected finally with live virus (without adjuvant) at the last week. Two weeks after the last injection bleeding was carried out from the goat. The blood was allowed to clot at room temperature and spun at 1000 rpm for 5 min, hyperimmune serum was harvested, the antibody titer was determined by ELISA. The HIS showing the highest antibody titer was aliquoted and distributed in 1 mL quantity stored at − 20 °C until further required. Therefore, this HIS was employed in the ELISA test as a positive reference sera while pre-immune sera collected from experimental animal used as negative control.

### Serological screening and assay procedures

#### Orf virus antigen and determination of Orf virus total protein concentration

The serological screening was done using an in-house developed antigen-coated ELISA. The virus antigen used for the coating of ELISA plate was a local orf virus isolate (UPM1/14) obtained from the Virology Laboratory, Faculty of Veterinary Medicine of the University Putra Malaysia from previous outbreak cases of contagious ecthyma [10]. This virus was propagated in lamb testicle cell (LT) monolayers as described by Bala et al. [27] and Abdullah et al. [10]. Upon propagation, the virus was concentrated in polyethylene Glycol (PEG) and purified in a cushion of 36% sucrose gradient and 10–50% sodium diatrizoate gradient [81]. The virus pellet obtained was reconstituted in sterile phosphate buffered saline pH 7.4 and kept at -70 °C until required. The total protein concentration of purified Orf virus was determined using Bradford assay method, total Orf virus concentration in the virus solutions was determine with aid of prism 5 software by plotting the protein concentration against the corresponding absorbance to obtain a standard curve.

#### Optimization of ELISA reagents

The working concentrations for Orf antigen, conjugate and antibodies were optimized using standard protocol of chequerboard titration adopted from Babiuk et al., [82]; Bhanuprakash et al., [83]; Niang, [84]; Azmi and Field, [85] with some minor changes. The positive and negative control sera obtained from post-vaccinated and pre-vaccinated animals were tested using a two-fold dilution. The optimal dilution the Orf virus antigen and reference HIS positive sera were selected using the antigen and serum dilutions that gave maximal difference in reading of the absorbance between positive and negative sera.

#### ELISA procedure

An enzyme linked immunosorbent assay (ELISA) was developed in-house as described by Azmi and Field [85] with minor modifications. All reagents including conjugate, substrate, buffers and washing procedures of plates were prepared according to standard procedures. ELISA test procedure was conducted by an initial coating of the 96-well plate specially designed for use in ELISA assays (Dynatech Immunolon, USA) [86]. The working volume of each of the reagents was 50 μl. Purified orf virus antigen was diluted in sodium hydrogen carbonate (NaHCO_3_) buffer (pH 9.6) to give a final concentration of 10 μg/ml antigen protein, and 50 μl of this was used for coating of the plate and then incubated at 4 °C overnight. Following overnight incubation, the plate was washed three times with Phosphate Buffered Saline Tween-20 (PBST), the washing was carried out manually by filling each of the well with 200 μl of the washing buffer, with the aid of a multi-channel micropipette, and then allowed to stand for a few seconds before discarding. This wash procedure was repeated 3 times. After washing 50 μl of 2% of Fraction V (Bovine Serum Albumin (BSA)) (Sigma, UK) was added and the plate was incubated at 45 °C for 2 h, in order to block any unspecific unbound antigens. Upon completion of the incubation, the plate was again washed three times with PBST-tween-20. A two-fold dilutions of the test sera were added to the plate and incubated at 37 °C for 1 h before the plate was washed 3 times using the same wash buffer. A pre-diluted rabbit anti-goat (for goat serum) and anti-sheep (for sheep serum) peroxidase conjugated immunoglobulin G (KPL, USA) was added and allowed to react with the antigen bound goat/sheep antibodies by incubation at 37 °C for 1 h. The plate was then washed 3 times and the substrate 2-2^l^ – Azino-bis (3-ethylbenzthioline − 6-sulfonic acid) ABTS diluted in citrate phosphate buffer (containing 0.01 30% hydrogen peroxide (H_2_O_2_)) was added and the plate was incubated at room temperature for 30 min. At the end of the incubation period, the optical density was read at 450 nm in an ELISA reader (TECAN Infinite M200).

#### Determination of cut off value

The value for ELISA cut-off threshold for tested samples at an optimized dilution of all the reagents was determined by taking the mean absorbance (O.D) reading of pre-immune negative sera plus three standard deviations [36, 82] (mean + S.D.; mean = 0.17; S.D. = 0.097, three times S.D. = 0.291 therefore CV is equal to 0.461). Any sample(s) with O. D reading above this CV was considered as positive for anti-Orf virus antibodies. Therefore, all the O.D. readings obtained from sera of animals were interpreted as positive when the value is greater than the cut-off value or deduced as negative if the value is otherwise.

#### Testing for cross-reactivity of ELISA assay

As a means of quality control of this in-house ELISA and in order to rule out any cross reactivity of this ELISA assay with similar virus of small ruminants, a panel of positive sera for Blue tongue (BTV) and Schemallenburg (SBV) viruses were tested using this developed ELISA. This positive BTV and SBV sera samples were available in the virology laboratory of the Universiti Putra Malaysia which were initially collected from confirmed disease cases occurring among some farms.

#### Sensitivity and specificity of the ELISA assay

The sensitivity and specificity of the ELISA employed in this study was determined based on the true positive and false negative value subjected to analysis and interepreted from the optimal cut-off point described elsewhere [36, 82, 83]. Both sensitivity and specificity values were calculated using Receiver Operating Characteristics (ROC) curves with the aid of MedCalc software (MedCalc Statistical Software version 18.11 (MedCalc Software bvba, Ostend, Belgium; http://www.medcalc.org; 2018).

### Data analysis

Information procured for both Sections A and B of the questionnaires was incorporated into Microsoft Office Excel and analysed using JMP software. The likelihood ratio and regression model were used to evaluate the farmer’s compliance level towards HHP. Responses to each sub-unit questions of the 14 major components modules of HHP (Table 8) were expressed as percentage to indicate overall farmers’ compliance level and their association determined after subjecting the result to chi-squre analysis with the aid of JMP Statistics software (SAS Campus Drive, USA). Similarly, all the data obtained from section B (demography and risk factors for exposure of individual animals) as well as the ELISA results were incorporated into the JMP software version 14, and analysed for prevalence rate and association of each risk factor using chi-square test.

## Additional file

Additional file 1: Questionnaire. (DOCX 59 kb)

## Data Availability

The datasets generated and/or analysed in the present study are not available to the public since it belongs to the Universiti Putra Malaysia, however, data can be made available upon request by contacting Prof. Dr. Mohd Azmi Mohd Lila via email azmi@upm.edu.my.

## Abbreviations

ABTS: 2-2^l^ – Azino-bis (3-ethylbenzthioline − 6-sulfonic acid)
BSA: Bovine Serum Albumin
DVS: Department of Veterinary Services
ELISA: Enzyme Linked Immunosorbent Assay
H_2_O_2_: Hydrogen peroxide
HHP: Herd Health Program
IACUC: Institutional Animal Care and Use Committee
MARDI: Malaysian Agricultural Analysis and Development Institute
MESTECC: Ministry of Energy, Science, Technology, Environment & Climate Change
MOSTI: Ministry of Science, Technology and Innovation Sciencefund
NaHCO_3_: Sodium Hydrogen Carbonate
O.D: Optical Density
PBST: Phosphate Buffered Saline Tween 20
PEG: Polyethylene Glycol
rpm: Revolution Per Minute
UVH: University Veterinary Hospital
X^2^: Chi Square
