# Supplementary material for: An association of Orf virus infection among sheep and goats with herd health programme in Terengganu state, eastern region of the peninsular Malaysia
Source: BMC Vet Res. 2019 Jul 18;15:250. doi: 10.1186/s12917-019-1999-1 (PMC6639921; doi:10.1186/s12917-019-1999-1)
Supplement: Supplementary file 1 — Questionnaire. (DOCX 59 kb) [file 12917_2019_1999_MOESM1_ESM.docx]

**QUESTIONNAIRE**

**Section A: General Farm Management Practice**

1. Name of the farm……………………………………………………………………………………..

2. Location of the farm (Address): ……………………………………………………………………..

3. Main activity of the farm: ……………………………………………………………………………

4. Area of pasture: ………………………………………………………………………………………

5. Total animals: ………………………………………………………………………………………..

6. Number of new stocks: ………………………………………………………………………………

7. Animal’s identification (Ear tagging, etc): ……………………………………………..……………

8. Type of farming: Sheep only ( ) Goat only ( ) Mixed ( )

9. Management: Intensive ( ) Semi intensive ( ) Extensive ( )

10. Type of feeding: ……………………………………………………………………………………

11. Degree of infestation with weeds that can cause injury (thistle, nettle, dock and ragwort):

Low ( ) Medium ( ) High ( )

12. Lambing management: Indoor ( ) Outdoor ( )

13. History for viral disease? Yes ( ) No ( )

1. FMD
2. BVDV
3. Goat pox
4. Orf virus

14. Percentage of lambs and ewes in the flock affected with Orf…………………………………….…

15. Management of animals affected with Orf disease

( ) Culling ( ) Medication at farm ( ) Take to nearest clinic ( ) Others…………………………..

16. Medication given (if applicable) ....…………………………………………………………………

17. Has this farm ever practice autogenous vaccination against Orf?

Yes ( ) No ( ) If yes, specify…………………………………………………………………….…………………

18. Vaccination of ewes and/or lambs against

1. Orf Yes ( ) No ( )
2. Other viral disease Yes ( ) No ( ) If yes, specify……………………….………………….………………………………………
3. Bacterial disease Yes ( ) No ( ) If yes, specify…………………………………………………………………………………..

19. Vaccination status?

Up to date ( ) Only during outbreak ( )

20. Personnel who administered vaccine: Farmer ( ) Veterinarian ( ) Others ( )……………………

21. Any abnormal skin disease observed among the workers?

Yes ( ) if yes, specify the location No ( )

Remarks

…………………………………………………………………………………………………………

…………………………………………………………………………………………………………

**Section B: Farm’s Herd Health Program Compliance**

| 1. Do you know what herd health program is? 2. Are you aware of herd health program existence? | Yes No  Yes No |
| --- | --- |

1. If you are aware, please tick ( / )programs included in herd health program:

| **Program** | **Yes** | **No** |
| --- | --- | --- |
| 1. Housing condition (eg : roof, flooring, ventilation, sanitation) |  |  |
| 1. Feed and feeding management (feed storage, amount of feed required per animal) |  |  |
| 1. Deworming / Deticking |  |  |
| 1. Vaccination program |  |  |
| 1. Farm biosecurity |  |  |
| 1. Waste disposal ( eg : dead animal/ manure) |  |  |
| 1. Fly , pest and odour control (eg: rat) |  |  |
| 1. Milking management |  |  |
| 1. Reproductive management (eg: estrus synchronization, soundness examination |  |  |
| 1. Lamb/kid management |  |  |
| 1. Doe/ewe management |  |  |
| 1. Animal identification |  |  |
| 1. Drug management (record system, storage) |  |  |
| 1. Disease monitoring program ( routine sampling for laboratory diagnosis) |  |  |

1. Where do you learn about the herd health program (can tick (/) one only)

| **Sources** |  |
| --- | --- |
| Course offered by Department of veterinary service (DVS) |  |
| Private veterinarian |  |
| UPM Ladang angkat program |  |
| Internet |  |
| Other farmer |  |

| 1. Are you practising herd health program? 2. If yes, do you think that you are practising herd health program properly? 3. Please tick (/) which one did you practiced? | | | | | | | | | | | Yes No  Yes No | | | | | | | | | | | |
| --- | --- | --- | --- | --- | --- | --- | --- | --- | --- | --- | --- | --- | --- | --- | --- | --- | --- | --- | --- | --- | --- | --- |
| **a. Housing condition (sanitation) H1 to H8** | | | | | | | | | | | | | | **Yes** | | | | | **No** | | | |
| Is your farm located in a free disease area? | | | | | | | | | | | | | |  | | | | |  | | | |
| Did you do any research/get consultation about the location before setting up your farm? | | | | | | | | | | | | | |  | | | | |  | | | |
| Do you think that the goat/sheep house is adequately ventilated? | | | | | | | | | | | | | |  | | | | |  | | | |
| Do you know hot environment causes heat stress in goat/sheep thus reduces milk production? | | | | | | | | | | | | | |  | | | | |  | | | |
| Do you think the floor is safe and not slippery for the goat/sheep? | | | | | | | | | | | | | |  | | | | |  | | | |
| Do you provide beddings (eg: rubber mat) for your goat/sheep? | | | | | | | | | | | | | |  | | | | |  | | | |
| How many times do you wash the floor per day? (circle the answer) | | | | | | | | | | | | 1 | | 2 | | | | | 3 | | | |
| How many times do you wash feed and drinking trough per month? Please state:________________ | | | | | | | | | | | | | | | | | | | | | | |
| **b. Feed and feeding management F1 to F7** | | | | | | | | | | | | | | | **Yes** | | | | | **No** | | |
| Do you store feed properly in closed container to prevent from any possible contamination such as pest or fungi? | | | | | | | | | | | | | | |  | | | | |  | | |
| Do you make sure that your pasture/grass does not has access from outside ruminant animal? | | | | | | | | | | | | | | |  | | | | |  | | |
| Do you know that harvesting grass at different growth stage contain different amount of nutrient? | | | | | | | | | | | | | | |  | | | | |  | | |
| Are you aware that different growth stage / function of animal have different nutrient requirement? | | | | | | | | | | | | | | |  | | | | |  | | |
| Did you give any feed supplement? (please tick (/) :  Mineral block ( ) multivitamin injection ( ) please state : ______________________________ | | | | | | | | | | | | | | | | | | | | | | |
| Do you do: i) feed analysis on grass and/or pellet? Yes No ii) Soil quality? Yes No | | | | | | | | | | | | | | | | | | | | | | |
| **c. Parasite control program** | | | | | | | | | | | | | | | **Yes** | | | | | **No** | | |
| **i) deworming program P1 to P7** | | | | | | | | | | | | | | | | | | | | | | |
| Do you deworm your goat/sheep? | | | | | | | | | | | | | | |  | | | | |  | | |
| Who did the deworming? (Circle your answer) : yourself / your worker / vet / dvs | | | | | | | | | | | | | | | | | | | | | | |
| **Answer these questions below, If you or your worker did the deworming** | | | | | | | | | | | | | | | | | | | | | | |
| When did you start to deworm? (weeks old) ( circle your answer) : 4 / 6 / 8 / 10 / 12 / 16 / 18 weeks | | | | | | | | | | | | | | | | | | | | | | |
| How frequent did you deworm? (circle your answer) :  depend on fecal egg count or worm burden / every 3 months / other: __________months | | | | | | | | | | | | | | | | | | | | | | |
| Did you check the expiry date of the drugs before use? | | | | | | | | | | | | | | |  | | | | |  | | |
| Did you make sure the dose and volume given is correct? | | | | | | | | | | | | | | |  | | | | |  | | |
| Do you know that giving inadequate dose of dewormer can cause drug resistance? | | | | | | | | | | | | | | |  | | | | |  | | |
| Do you know that alternate use of different dewormer can reduce drug resistance? | | | | | | | | | | | | | | |  | | | | |  | | |
|  | | | | | | | | | | | | | | | | | | | | | | |
| **ii) Deticking program D1 to D3** | | | | | | | | | | | | | | | **Yes** | | | | | **No** | | |
| Do you detick your goat/sheep? | | | | | | | | | | | | | | |  | | | | |  | | |
| Who does the deticking? (Circle your answer) : yourself / your worker / vet / dvs | | | | | | | | | | | | | | |  | | | | |  | | |
| How frequent do you detick? (circle your answer) :  every 3 month / depend on tick burden / other: ____________ | | | | | | | | | | | | | | |  | | | | |  | | |
| **d. Vaccination program** V1 to V3 | | | | | | | | | | | | | | | **Yes** | | | | | **No** | | |
| Do you vaccine your goat/sheep? | | | | | | | | | | | | | | |  | | | | |  | | |
| Who did the vaccination? (Circle your answer) : yourself / your worker / vet / dvs | | | | | | | | | | | | | | | | | | | | | | |
| Do you know the vaccination protocol? (at what age to start vaccine and booster) | | | | | | | | | | | | | | |  | | | | |  | | |
| **e. Farm biosecurity** B1 to B7 | | | | | | | | | | | | | | | **Yes** | | | | | **No** | | |
| Does your farm area (including pasture area) secured by fence? | | | | | | | | | | | | | | |  | | | | |  | | |
| If yes, do you always check the fencing is in good and safe from outside animal? | | | | | | | | | | | | | | |  | | | | |  | | |
| Do you emphasize on using uniform and/or gum boot every day? | | | | | | | | | | | | | | |  | | | | |  | | |
| Do you have isolation pen in the farm? | | | | | | | | | | | | | | |  | | | | |  | | |
| Do you isolate sick and new animal separately in different isolation pen? | | | | | | | | | | | | | | |  | | | | |  | | |
| **Please tick (/) which one do you use:** | | | | | | | | | | | | | | | | | | | | | | |
| Vehicle disinfection | | | Manual spray | | | | | | | | | | | | | | | | |  | | |
|  |  |  | Manual high pressure spray | | | | | | | | | | | | | | | | |  | | |
|  |  |  | Open wheel dip | | | | | | | | | | | | | | | | |  | | |
|  |  |  | Covered wheel dip | | | | | | | | | | | | | | | | |  | | |
|  |  |  | Covered wheel dip with spray | | | | | | | | | | | | | | | | |  | | |
| Footbath to the  Cattle house | | | Randomly place | | | | | | | | | | | | | | | | |  | | |
|  |  |  | Entrance to every house | | | | | | | | | | | | | | | | |  | | |
| **f. Waste disposal W1 TO W5** | | | | | | | | | | | | | **Yes** | | | | | **No** | | | | |
| Do you dispose farm waste properly | | | | | | | | | | | | |  | | | | |  | | | | |
| **How do you dispose dead sheep/goat ?(please tick (/)** | | | | | | | | | | | | | | | | | | | | | | |
| Incinerator | | Burn | | | Pit | | | | Bury | Other | | | | | | | | | | | | |
|  | |  | | |  | | | |  |  | | | | | | | | | | | | |
|  | | | | | | | | | | | | | | | | | | | | | | |
| **How do you dispose the goat/sheep manure ?(please tick (/)** | | | | | | | | | | | | | | | | | | | | | | |
| Just flush with water to push it out from goat/sheep house | | Flush with water and flow it into waste pond | | | Flush with water and let it flow into water stream or river | | | | Collect and sell in wet form | Collect and sell in dried form | | | | | | | | | | | | |
|  | |  | | |  | | | |  |  | | | | | | | | | | | | |
| If the flushed water flow into river, do you treat the water first? Yes No | | | | | | | | | | | | | | | | | | | | | | |
| Is there any water quality inspection done before the water flow into river? Yes No | | | | | | | | | | | | | | | | | | | | | | |
| **g. Fly, pest and odour control C1 to C2** | | | | | | | | | | | | | | | | | | | | | | |
| Does your farm have problem with(please tick (/) : fly pest odour | | | | | | | | | | | | | | | | | | | | | | |
| **How do you control the problem? (please tick (/)** | | | | | | | | | | | | | | | | | | | | | | |
|  | **Chemical use** | | | | | | **Other methods:** | | | | | | | | | | | | | | | |
| **Fly** | Larva stage  Adult stage | | |  | | |  | | | | | | | | | | | | | | | |
| **Pest** |  | | |  | | |  | | | | | | | | | | | | | | | |
| **Odour** |  | | | | | |  | | | | | | | | | | | | | | | |
| **h. Milking management (mastitis control program) M1 to M4** | | | | | | | | | | | | | **Yes** | | | | | **No** | | | | |
| Do you make sure proper installation and function (suction pressure) of the milking machine? | | | | | | | | | | | | |  | | | | |  | | | | |
| Do you make sure the milking area and doe’s/ewe’s udder were cleaned before milking process? | | | | | | | | | | | | |  | | | | |  | | | | |
| Do you perform California mastitis test (CMT)at least once a month before milking? | | | | | | | | | | | | |  | | | | |  | | | | |
| How you wash the milking machine after milking? Hot water Disinfect chemically | | | | | | | | | | | | | | | | | | | | | | |
| *milk storage?**i. Reproductive management R1 to R15** | | | | | | | | | | | | | | | | **Yes** | | | | | **No** | |
| Are you aware that keeping accurate reproductive record is very important to improve animal performance? ( eg: kidding/lambing rate, AI success rate) | | | | | | | | | | | | | | | |  | | | | |  | |
| Do you observe for passage of placenta and remove it from kidding/lambing area? | | | | | | | | | | | | | | | |  | | | | |  | |
| Do you know that goat/sheep should not be allowed to eat placenta because it will not digested? | | | | | | | | | | | | | | | |  | | | | |  | |
| Does your farm perform? estrus synchronization and artificial insemination( ) or natural mating ( ) | | | | | | | | | | | | | | | | | | | | | | |
| Do you carry out buck/ram soundness examination before buying a breeder buck/ram? | | | | | | | | | | | | | | | |  | | | | |  | |
| Do you perform routine hoof care to buck/ram and doe/ewe to facilitate in mating process? | | | | | | | | | | | | | | | |  | | | | |  | |
| **Please tick (/) which one do you kept proper record** | | | | | | | | | | | | | | | | | | | | | | |
| 1) Kidding/lambing date | | | | | |  | | 6) On heat date | | | | | | | | | | | | |  | |
| 2) Kidding/lambing difficulties | | | | | |  | | 7) Mating date | | | | | | | | | | | | |  | |
| 3) Retained placenta | | | | | |  | | 8) buck/ram and doe/ewe used | | | | | | | | | | | | |  | |
| 4) Abortion | | | | | |  | | 9) Medication and hormonal treatment | | | | | | | | | | | | |  | |
| 5) Abnormal vaginal discharge | | | | | |  | |  | | | | | | | | | | | | |  | |
| **j. Kid/lamb management** K1 to K14 | | | | | | | | | | | | | | | | | **Yes** | | | | | **No** |
| Do you make sure that pregnant doe/ewe which are almost due to kidding/lambing get intensive monitoring for any assistant during calving? | | | | | | | | | | | | | | | | |  | | | | |  |
| Do you make sure the doe/ewe lick the kid/lamb to dry? | | | | | | | | | | | | | | | | |  | | | | |  |
| Do you make sure that the kid/lamb can stand up and get colostrum within 6 hours after born? | | | | | | | | | | | | | | | | |  | | | | |  |
| Do you trim navel if it is longer than 6 cm and then dip the navel with tincture iodine? | | | | | | | | | | | | | | | | |  | | | | |  |
| Do you tag or tattoo the kid/lamb within 1 week old? | | | | | | | | | | | | | | | | |  | | | | |  |
| Please state age range if you does not raise kid/lamb until adult but sell it : _________ until ______ | | | | | | | | | | | | | | | | | | | | | | |
| Do you practice FMD and HS vaccination after 3 months old followed by the booster? | | | | | | | | | | | | | | | | |  | | | | |  |
| **Did you properly record these: (please tick (/) which did you record** | | | | | | | | | | | | | | | | | | | | | | |
| Birth weight ( ) Birthday ( ) Tag no.( ) Buck/ram tag no. ( ) Doe/ewe tag no. ( ) Breed ( ) Sex ( ) | | | | | | | | | | | | | | | | | | | | | | |
|  | | | | | | | | | | | | | | | | | | | | | | |
| **k. Doe/ewe management E1 to E5** | | | | | | | | | | | | | | | | | **Yes** | | | | | **No** |
| Do you gather all the does/ewes which had just delivered together in a group to facilitate in examination for problems (eg: retained placenta, endometirits etc) in the future? | | | | | | | | | | | | | | | | |  | | | | |  |
| Do you group doe/ewe according to function?(eg: on heat (waiting for service), pregnant, lactating, dry etc) | | | | | | | | | | | | | | | | |  | | | | |  |
| Do you give extra supplement (eg: concentrates, molasses etc) to doe/ewe in late pregnancy? | | | | | | | | | | | | | | | | |  | | | | |  |
| At which month of pregnancy do you dry the doe/ewe? ________ months | | | | | | | | | | | | | | | | |  | | | | |  |
| How long do you dry the doe/ewe ________ months? | | | | | | | | | | | | | | | | |  | | | | |  |
| **l. Animal identification T1 to T4** | | | | | | | | | | | | | | | | | **Yes** | | | | | **No** |
| Did you perform tagging to all new animals? | | | | | | | | | | | | | | | | |  | | | | |  |
| Did you encounter problem of loss tag in animal? | | | | | | | | | | | | | | | | |  | | | | |  |
| If yes, do you re-tag? | | | | | | | | | | | | | | | | |  | | | | |  |
| Do you agree that identification assist in record keeping? | | | | | | | | | | | | | | | | |  | | | | |  |
| **m. Medication/Drug management (record system, storage) G1 to G4** | | | | | | | | | | | | | | | | | **Yes** | | | | | **No** |
| Do you really know the indication, uses and storage of medication/drug that you kept? | | | | | | | | | | | | | | | | |  | | | | |  |
| Do you get any consultation from licensed veterinarian first before buy and use it? | | | | | | | | | | | | | | | | |  | | | | |  |
| Do you check the drug bottle/box for withdrawal period and expiry date? | | | | | | | | | | | | | | | | |  | | | | |  |
| Do you have medication/drug recording system(date given, drug dosage, route, volume) | | | | | | | | | | | | | | | | |  | | | | |  |
| **n. Disease monitoring program X1 to X5** | | | | | | | | | | | | | | | | | **Yes** | | | | | **No** |
| Is there any history of disease outbreak in your farm | | | | | | | | | | | | | | | | |  | | | | |  |
| If yes, please state what and when? i)  ii) | | | | | | | | | | | | | | | | | | | | | | |
| Do you request for post mortem examination if there is death case? | | | | | | | | | | | | | | | | |  | | | | |  |
| Do you request for routine disease monitoring?(eg: brusellosis, Johnes, blood parasitism) | | | | | | | | | | | | | | | | |  | | | | |  |
| Did your farm ever do antibiotic sensitivity test? | | | | | | | | | | | | | | | | |  | | | | |  |

1. Practising herd health program properly will:

|  | Yes | | No |  |
| --- | --- | --- | --- | --- |
| Increase farm productivity |  | |  |  |
| Increase farmer income |  | |  |  |
| Reduce treatment cost |  | |  |  |
| Reduce animal mortality |  | |  |  |
| Increase animal growth rate/body weight |  | |  |  |
| Increase milk production |  | |  |  |
| Reduce stress in animal |  | |  |  |
| Increase animal immunity |  | |  |  |
| Prevent disease introduction into farm |  | |  |  |
| Prevent disease transmission between animal in farm |  | |  |  |
| Prevent disease outbreak |  | |  |  |
| Prevent zoonotic disease transmitted from animal to human |  | |  |  |
| 1. Are you willing to invest money for herd health program that guarantee long term profitability in future? 2. Do you prefer to avoid practicing herd health program and just spend money for treatment cost, disease control cost and mortality cost? | | Yes No  Yes No | | |

**Section C: Demography and risk factor for individual animal’s exposure**

**Sample ID……………………..**

1. Date: ………………………….……………………………………………………..
2. Animal details: Goat ( ) Sheep ( )
3. Animal ID: …………………………………….…….……………………………...
4. Sex: Male ( ) Female ( )
5. Age: …………………….………………………….…………….………………….
6. Type of breed: ………………………………………………………………………
7. Physical injury: Yes ( ) Site…………………No ( )……………………………..
8. Does the animal present with any sign/lesion of Orf?

Yes ( ) specify …………….…………No ( )

1. Is the animal newly introduced (< 3 months) into the farm?

Yes ( ) No ( )

Remarks:

………………………………………………………………………………………

………………………………………………………………………………………

………………………………………………………………………………………
